# Arthrogryposis in infancy, multidisciplinary approach: case report

**DOI:** 10.1186/1471-2431-13-184

**Published:** 2013-11-11

**Authors:** Anna Binkiewicz-Glinska, Agnieszka Sobierajska-Rek, Stanislaw Bakula, Jolanta Wierzba, Konrad Drewek, Ireneusz M Kowalski, Katarzyna Zaborowska-Sapeta

**Affiliations:** 1 Department of Rehabilitation, Medical University of Gdansk, ul. Debinki 7, 80-952 Gdańsk, Poland; 2 Department of General Nursing University of Gdansk, 80-952 Gdańsk, Poland; 3 Department of Orthopedics and Traumatology, Medical University of Gdansk, ul. Powstancow Warszawskich 1 / 2, 80-152 Gdansk, Poland; 4 Department of Rehabilitation, University of Warmia and Mazury in Olsztyn, ul. Oczapowskiego 2, 10-719 Olsztyn, Poland

**Keywords:** Infant, Arthrogryposis, Rehabilitation

## Abstract

**Background:**

Arthrogryposis multiplex congenita is an etiopathogenetically heterogeneous disorder characterised by non-progressive multiple intra-articular contractures, which can be recognised at birth. The frequency is estimated at 1 in 3,000 newborns. Etiopathogenesis of arthrogryposis is multifactorial.

**Case presentation:**

We report first 26 weeks of life of a boy with severe arthrogryposis. Owing to the integrated rehabilitation approach and orthopaedic treatment a visible improvement in the range of motion as well as the functionality of the child was achieved. This article proposes a cooperation of various specialists: paediatrician, orthopaedist, specialist of medical rehabilitation and physiotherapist.

**Conclusions:**

Rehabilitation of a child with arthrogryposis should be early, comprehensive and multidisciplinary. Corrective treatment of knee and hip joints in infants with arthrogryposis should be preceded by the ultrasound control. There are no reports in the literature on the ultrasound imaging techniques which can be used prior to the planned orthopaedic and rehabilitative treatment in infants with arthrogryposis. The experience of our team indicates that such an approach allows to minimise the diagnostic errors and to maintain an effective treatment without the risk of joint destabilisation.

## Background

Arthrogryposis multiplex congenita (AMC) is an etiopathogenetically heterogeneous disorder, characterised by non-progressive multiple intra-articular contractures, which can be recognised at birth [1]. The frequency is estimated at 1 in 3,000 newborns [2]. Etiopathogenesis of arthrogryposis is multifactorial.

Symptoms of some forms of arthrogryposis can be found in selected monogenic diseases (with the autosomal recessive, autosomal dominant or X-linked inheritance), chromosomal aberrations and syndromes of multi-organ congenital malformations.

Arthrogryposis can also result from environmental factors, affecting the mother and the foetus, such as: infections, medications, traumas, chronic illnesses, oligohydramnios or abnormal structure of the uterus [3]. These are, however, factors leading not only to arthrogryposis, but underlying about 7% of all congenital malformations [4]. In the last months of pregnancy reduction of foetal movements is a common denominator of these cases. Movement is essential for the normal development of joints and the periarticular tissues. Lack, or limitation, of movements leads to excessive development of the periarticular connective tissue [5]. Contractures secondary to foetal akinesia are usually more severe in infants diagnosed in early pregnancy [6].

Only few reports can be found in the literature on the rehabilitation programmes for infants with AMC. Paediatricians are usually the primary care doctors for children with AMC, later, often at the time the children start to walk, they are usually referred to orthopaedists [7].

This study presents a multi-disciplinary rehabilitation approach in a case of a child with full-blown form of arthrogryposis, complicated by perinatal hypoxia. We propose a cooperation of specialists of various disciplines, initiated as early as the neonatal period.

## Case presentation

### Case history

The boy was born, after the first pregnancy, to young, healthy, unrelated parents. In the first weeks of the pregnancy X-rays of the head and palm were taken in the mother because of the injury. The mother had been taking NSAIDs until the 5^th^ week of gestation. It was not until the 5^th^ week that the pregnancy was detected. Progesteron was recommended vaginally because of the spotting. Furthermore, starting from the 8^th^ week, clotrimazole and vitamins (Femibion) were used throughout the pregnancy. No signs of deformation within the joints were reported in the routine clinical ultrasound evaluation but the oligohydramnios observed in 33th week of pregnancy was linked with the signs of foetus poor movement described by the mother as “very calm baby” during the last weeks of the pregnancy. A boy was delivered after spontaneous labour at term (40^th^ week of gestation) in a general poor condition (Apgar score of 1, 5 and 6 in the - 1^st^, 3^rd^ and 5^th^ minutes respectively) with the birth weight of 4150 g, length 56 cm and head circumference 36 cm. The child required ventilation for 4 days. Antibiotics were administered, as an intrauterine infection was suspected based on elevated C-reactive protein levels. Seizures were observed in the first days of life, most likely resulting from the perinatal hypoxia. Subsequently some abnormal EEG readings were evident, requiring a short anticonvulsant therapy with phenobarbital.

**Figure 1:**
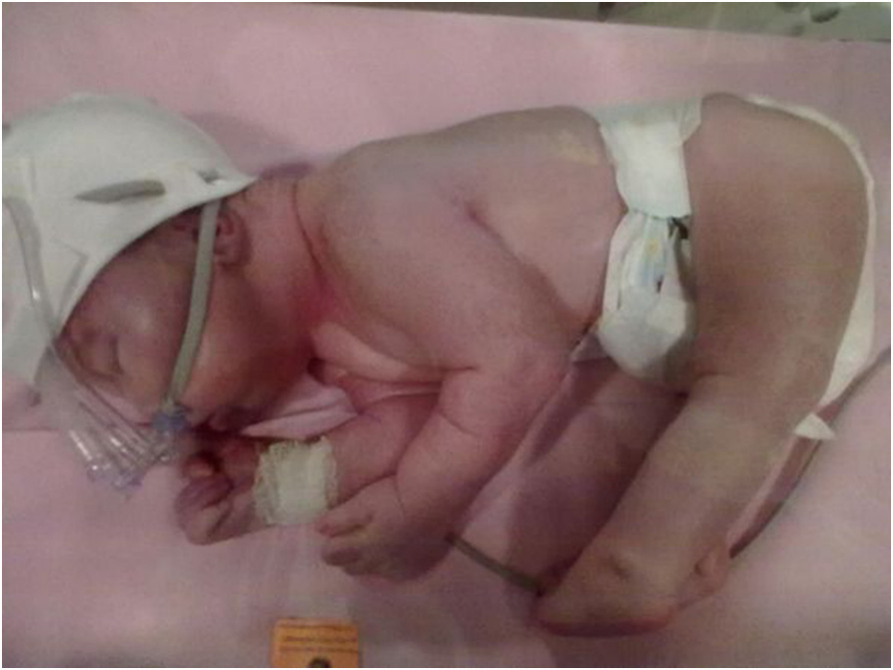
The first day of life.

**Figure 2:**
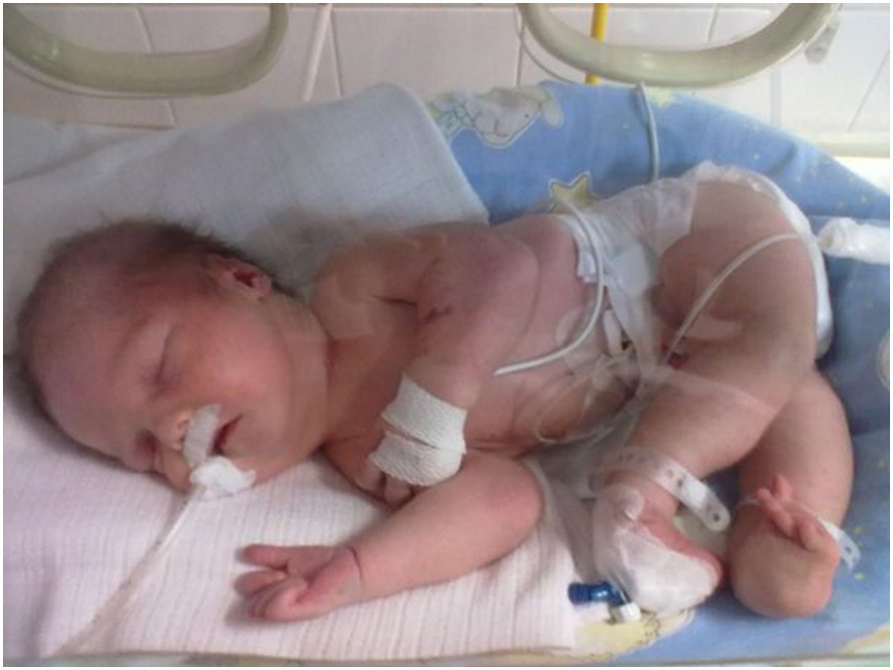
The third day of life – the whole figure – noticeable free thumbs and apparent dislocation on the left knee-joint.

Examination after birth revealed the presence of micrognathia, retrognathia and contractures of the joints typical for AMC: i.e. affecting the wrists, elbows and shoulders as well as legs in the form of clubfeet - scoring 4 on Dimeglio scale, accompanied by knee-joint subluxation and dislocation of the right hip joint (Figures 1 and 2). The baby presented no sucking reflex, a very weak reactivity to external stimuli, reduced muscle tone, especially in the head - shoulder line and weakened tendon reflexes. He stayed in a constrained position on the side.

**Figure 3:**
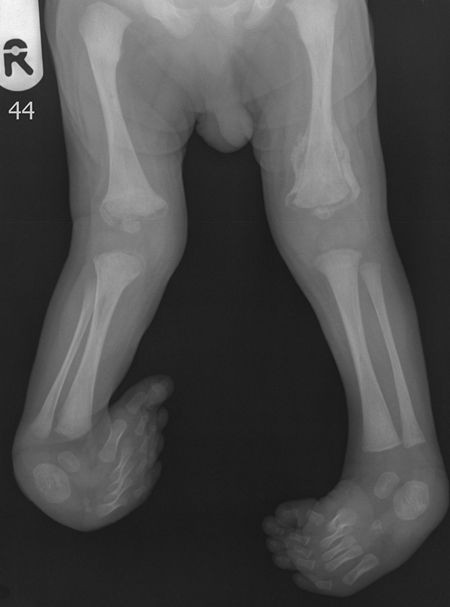
X-rays of the lower limbs taken at the age of 10 days.

An x-ray image of the lower limbs showed visible callus around the femur fractures, the occurrence of perinatal fracture and deformation typical for a clubfeet (Figure 3). An ultrasound examination of the hip and knee joints revealed the following: legs positioned in an internal rotation (90-100°), medial instability of both knees and the patella set to the medial side. No signs of hip dislocation were noted, with a good seating of the femoral head in the acetabulum and the correct depth of the sockets cover.

Cranial and abdominal ultrasound, as well as ophthalmic examination, revealed no pathology. Examination of the induced auditory potentials showed correct results from the brain stem and bilateral hearing loss at the level of 70 dB. Echocardiography revealed haemodynamically insignificant defect of the atrial septum (ASD). Due to the massive deformation of the occipital area, brain MRI was performed, detecting no abnormalities except for the 9 mm arachnoid cyst, adhering to the left temporal pole. The congenital infections - cytomegaly, toxoplasmosis, parvovirus infection, hepatitis type B and rubeola- were ruled out. Cytogenetic investigations presented normal male karyotype - 46, XY. Submicroscopic aberrations were excluded using microarray-based comparative genomic hybridisation (array-CGH) (array(1–22)×2,(X,Y)×1). Osteogenesis inperfecta was ruled out in further tests. EEG performed some months later showed no pathology.

More than one type of joints was affected. A very specific position with symmetric limb involvement was noted: arm-wrists, elbows and shoulders, fixed knees, equinovarus feet. Typical AMC inducing factors and symptoms could be identified in this case: X-rays were taken in the mother and NSAIDs were used until the 5^th^ week of gestation.

These could be the potential teratogenic agents in this case, but no signs of other major congenital disorders where found, cytogenetic investigations revealed no abnormalities. The clinical picture was not compatibile with any known monogenic disorder with AMC. The cause of oligohydramnios and the pathological reduction of the foetal movements had not been pinpointed. Bone fractures could only be explained by the difficulties during the labour. Perinatal asphyxia resulted in the seizures and perinatal hypotonia.

### Rehabilitation

Rehabilitation was initiated at the age of three weeks, the infant was retained in the hospital for eight weeks in order to stabilise the epileptic activity, to monitor nutrition (due to the lack of sucking reflex and dysphagia), and for the orthopaedic treatment.

The aim of the rehabilitation was to increase the range of motion in the joints and enhance the neuro-developmental stimulation.

The ultrasound was used to position the structures of the limbs. In particular, ultrasound imaging was helpful in positioning of the patella and the knee axis. The information obtained from ultrasound assessments allowed marking the structures on the skin. This simple procedure allowed for the progress of training, limiting, at the same time, the risk of the joint dislocation. Photographs of the training manouvers, with the marks on the skin, were taken over the therapy to instruct parents and other therapists.

Techniques used to increase the mobility in the joints:

– relaxing massage and stretching within the muscle attachments,

– articular mobilization techniques in strict accordance with the limits of mobility audited under the control of the ultrasound,

– corrective kinesiotaping on the dorsal side of the hand, stimulating straightening of the joints in the wrist and fingers,

– mobilization of the feet according to the Ponseti method.

Techniques of development stimulation

– the positional therapy,

– stimulation of the sensory systems, proprioceptive stimulation of the hand opening, techniques using the setting reflexes to stimulate supporting functions of the upper limbs, holding the head, etc.,

– sucking reflex stimulation.

Instruction for parents was an integral part of the rehabilitation: training in the laying position, placing, changing of positions, carrying, feeding, etc.

Careful and repeated training of the optimal axes in which the exercises, increasing the motion range, should be performed. Recording and photographing of the presented techniques was encouraged.

### Orthopaedic procedures

**Figure 4:**
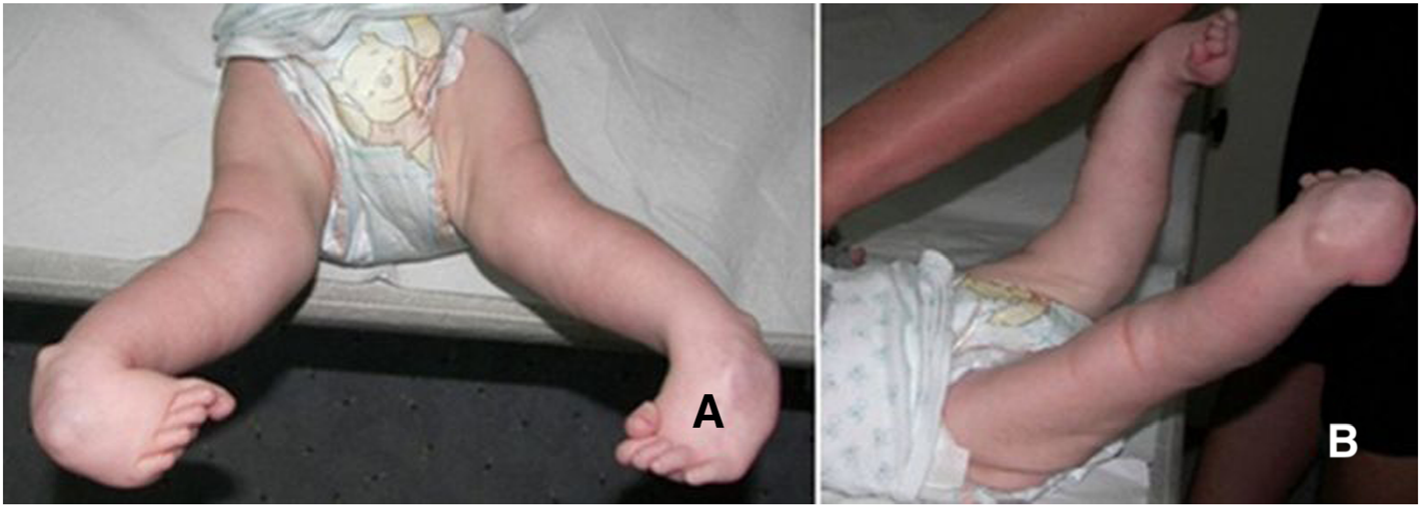
**Lower limbs at the age of 14 weeks.** Panel **A**: Front view. Panel **B**: Right side view.

Orthopaedic treatment was initiated at the age of 3 months . It started with a series of manipulative plasters using a modified Ponseti's protocol (treatment of clubfeet) (changed once a week, during the treatment intermissions the boy was taken for the rehabilitation at the ward). The condition before the plaster manipulation is presented at the Figure 4.

**Figure 5:**
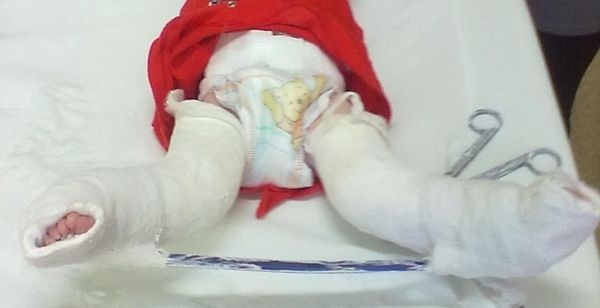
Manipulative casts – Improvement of knee flexion with bar improving external rotation in the hip joints, the first attempt to correct the foot by Ponseti – age 20 weeks.

**Figure 6:**
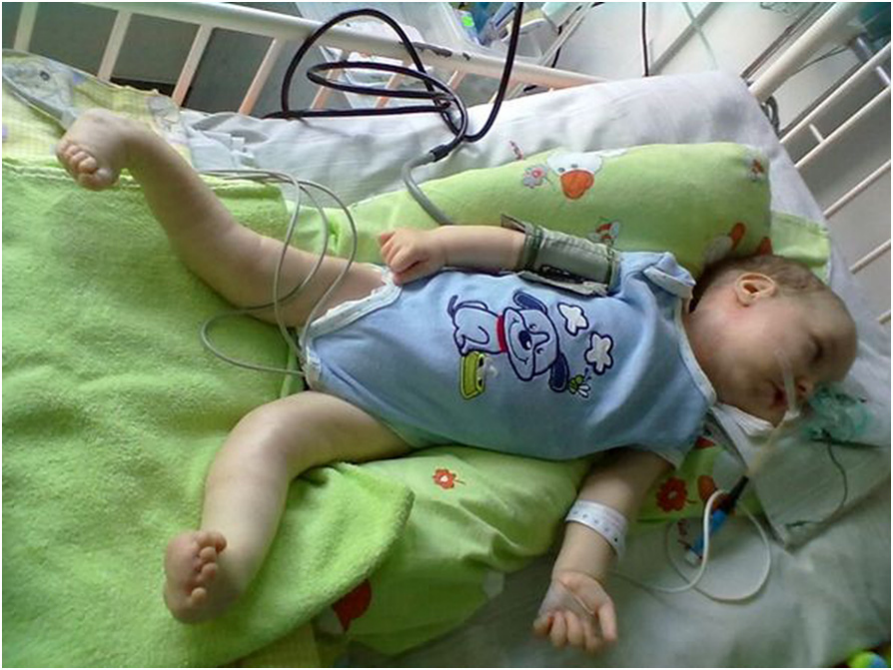
The improvement in the external rotation of the hip joints – age 20 weeks.

Through the use of plaster casts a correction of knee joint flexion to 45° was achieved, then plasters on the lower limbs were combined with an aluminium crossbar, in order to achieve a correction of the rotation in the hip joints (Figures 5 and 6).

Thanks to the integrated rehabilitation approach and orthopaedic treatment a visible improvement in the range of motion, as well as in the functionality of the child, was achieved. The child continues orthopaedic treatment and rehabilitation. Functional assessment and joint deformation assessment had been performed four times since the child’s birth (Table 1).

**Table 1:** Range of the joint mobility before and after rehabilitation.

	**Limited motion direction**	**Before rehabilitation (3 week of life)**	**After rehabilitation (26 week of life)**	**Improvement [°]**
**Shoulder joint L and R**	flexion	90°	170°	80°
	extension	20°	100°	80°
**Elbow joint L**	flexion	30°	120°	90°
**Elbow joint R**	straightening	−90°	−60°	30°
**Carpal joint L and R**	straightening	- 20°	stiffness	20°
	extension	−10°	stiffness	10°
**Hip joint L and R**	straightening	−115°	−45°	70°
	extension	20°	45°	25°
	external rotation	−135°	−115°	20°
**Knee joint L and R**	straightening	−15°	−5°	10°
	flexion	0°	45°	45°

## Discussion

Arthrogryposis in infancy is an interdisciplinary problem. Children affected by this disease require the care of paediatricians, rehabilitation professionals as well as orthopaedists.

Children with arthrogryposis can be subjected to orthopaedic treatment with satisfactory results. The current technological advancements allow for an orthopaedic interventions to minimise the limitations in everyday activities [8]. In the case discussed here, we dealt with a number of joint deformities. Limb axis distortion and significant contractures made it practically impossible to determine the position of each anatomical element in clinical assessment of the infant. Clinical observation suggested the presence of knee joint subluxations as well as hip joint dislocations. We found no reports in the literature on the ultrasound imaging techniques which can be used prior to the planned orthopaedic and rehabilitative treatment in infants with arthrogryposis. The experience of our team indicates that such an approach allows to minimise diagnostic errors and maintain an effective treatment without the risk of joint destabilisation.

The ultrasound revealed features of the medial instability of the knee joints resulting from the improper earlier rehabilitation by relatives during a short stay at home.

At the moment it is not possible to determine what level of motor and intellectual development the described child can reach [9]. Parallel occurrence of arthrogryposis, micrognathia, retrognathia, and the consequences of perinatal hypoxia make the prognosis difficult and uncertian. Intensive rehabilitation started early, supported by daily supervised exercises conducted by the parents gives the child with arthrogryposis an opportunity to improve the range of motion in the joints and to reduce the need for subsequent radical invasive corrections [10]. Treatment of clubfoot in arthrogryposis with Ponseti’s method is believed by many researchers to be an effective method helping to avoid radical surgery [11]. But despite the intensive rehabilitation and corrective actions the surgery for articular deformations may be unavoidable. In 76% of patients with arthrogryposis foot joints require surgical correction, knee joints need intervention in 39% of patients, and the hips in 18% of cases [10]. Surgical release of the soft tissues with the total release of the tendons is recommended before the child learns to walk. In the case described, a subtalar release treatment is planned as the use of plasters, and even release of the soft tissue in the case of arthrogryposis does not provide lasting results [11]. The literature emphasises the importance of the earliest possible initiation of the corrective action, with the best results obtainable in the first months of life [12]. While focusing on correcting the joint deformities, the child’s developmental stimulation should not be forgotten. Due to the articular restrictions an infant with arthrogryposis is less able to explore the world, thus the development of cognitive and motor function is hampered [13,14]. In case of infants with retrognathia the control of feeding is an important issue. Due to the sucking and swallowing disturbances it is extremely important to adjust the appropriate feeding method to avoid problems such as aspiration pneumonia or malnutrition.

Early rehabilitation of a child with arthrogryposis requires the involvement of the parents/guardians. Unfortunately, in the face of serious illness of the child very often there is a relationship crisis between the parents and even the disintegration of a relationship. Parents of a child burdened with severe congenital disease often experience disappointment, frustration and a sense of losing the control. The care for a sick child usually imposes changes of the everyday life, changes of the long-term life plans, which may result in depression and even in a post traumatic stress disorder [15].

## Conclusion

1. Rehabilitation of a child with arthrogryposis should be early, comprehensive and multidisciplinary.

2. Corrective treatment of knee and hip joints in the case of infants with arthrogryposis should be preceded by the ultrasound control.

## Consent

Written informed consent was obtained from the patient’s parents for publication of this Case report and any accompanying images. A copy of the written consent is available for review by the Editor of this journal.

## Competing interests

The authors declare that they have neither financial nor non-financial competing interests.

## Authors’ contributions

ABG – originator of the project in its final form, exercised rehabilitation consultations, commissioned rehabilitation activities, coordinated multidisciplinary team activities, obtained funds for the publication, translated the text from Polish and was responsible for drafting a cover letter. ASR – performed all rehabilitation activities, assessed the patient in terms of functionality and was responsible for collecting data and drafting the article. SB – substantial supervision. JW – was responsible for conducting the paediatric proceedings and edited the introduction to the article. KD – performed all consultations and orthopaedic actions, was consulted on the orthopaedic aspect of the article. IMK – made the final adjustments to the text and literature. KZS – introduced final modifications to the article and table. All authors approved the final version of the article.

## Authors’ information

ABG - MD, specialist of medical rehabilitation, member of Polish Rehabilitation Society, assistant in the Department of Rehabilitation.

ASR- Physiotherapist, PhD, assistant in the Department of Rehabilitation, member of Polish Physiotherapy Association.

SB - MD, associate professor in Medical University of Gdansk, specialist of internal medicine and medical rehabilitation, head of the Department of Rehabilitation, member of Polish Society of internal Medicine, member of Polish Rehabilitation Society, member of Rehabilitation, Physical Culture, Social Integration Committee of Polish Academy of Science.

JW - MD, PhD, specialist of paediatrics and clinical genetics, head of the Department of Infant Pathology, member of European and Polish Human Genetics Society, member of Polish Registry of Congenital Malformations Working Group, member of Advisory Council – Cornelia de Lange Syndrome Association.

KD - MD, PhD, specialist Of Orthopaedics, assistant in Department of Orthopaedics and Traumatology, member of Polish Orthopaedics and Traumatology Society, Polish Foot and Ankle Society and European Foot and Ankle Society.

IMK - MD, associate professor in University of Warmia and Mazury, specialist of paediatrics and medical rehabilitation, director of Rehabilitation Department, member of Polish Rehabilitation Society, voivodeship regional consultant of medical rehabilitation.

KZS - MD, specialist of medical rehabilitation, assistant in Department of Rehabilitation, member of Polish Rehabilitation Society and Polish Medical Writers Association.

## Pre-publication history

The pre-publication history for this paper can be accessed here:

http://www.biomedcentral.com/1471-2431/13/184/prepub
